# Condiciones socioeconómicas desfavorables asociadas a la presencia de obesidad y sus comorbilidades metabólicas en adultos de San Luis, Argentina

**DOI:** 10.31053/1853.0605.v80.n4.40737

**Published:** 2023-12-26

**Authors:** Stefano Staurini, Camila Niclis, María Silvina Calcagni, Darío Ramírez, Sandra Gómez-Mejiba, Laura R Aballay

**Affiliations:** 1 Universidad Nacional de Córdoba. Facultad de Ciencias Médicas. Escuela de Nutrición. Centro de Investigación en Nutrición Humana Córdoba Argentina; 2 Universidad Nacional de Córdoba. Facultad de Ciencias Médicas. Consejo Nacional de Investigaciones Científicas y Técnicas. Instituto de Investigaciones en Ciencias de la Salud Córdoba Argentina; 3 Universidad Nacional de San Luis. Facultad de Ciencias de la Salud. Carrera de Nutrición San Luis Argentina; 4 Universidad Nacional de San Luis. Consejo Nacional de Investigaciones Científicas y Técnicas. Laboratorio de Medicina Experimental y Traduccional San Luis Argentina

**Keywords:** sobrepeso, monitoreo epidemiológico, factores socioeconómicos, estilo de vida, enfermedades metabólicas, overweight, epidemiological monitoring, socioeconomic factors, life style, metabolic diseases, sobrepeso, monitoramento epidemiológico, fatores socioeconômicos, estilo de vida, doenças metabólicas

## Abstract

La prevalencia de obesidad depende de factores biopsicosociales y ambientales, y es un factor de riesgo para enfermedades transmisibles y no transmisibles. Objetivo: Determinar la asociación entre las características demográficas, socioeconómicas y del estilo de vida, y la presencia de obesidad y sus comorbilidades metabólicas (CM) en adultos de la ciudad de San Luis, Argentina.

Estudio observacional-poblacional-transversal en 306 individuos entre 18 y 85 años de San Luis, Argentina, seleccionados mediante un muestreo aleatorio multietápico, considerando una prevalencia de sobrepeso del 35% y un error de 0,05. Se indagaron variables socioeconómicas, demográficas, del estilo de vida, y se ajustaron modelos de regresión logística múltiple incluyendo presencia de obesidad y CM como variable de respuesta, y características sociodemográficas y del estilo de vida como covariables.

El 17,3% de los participantes presentó obesidad, un 3% diabetes, el 11% hipertensión arterial (HTA), el 3,3% dislipidemia y un 13% complicaciones isquémicas coronarias (CIC). La proporción de habitantes con al menos una de esas patologías fue del 26,8%. Un bajo Nivel de instrucción (NI) se asoció positivamente con la presencia de obesidad (OR 3,58; IC95% 1,04-12,24; p=0,04), y sus CM (OR 5,25; IC95% 1,05-26,23; p=0,04) respecto al NI alto. Asimismo, la posibilidad de presentar CIC se vio aumentada en personas con NI medio (OR 5,8; IC95% 1,12-30,19; p=0,03). Por otro lado, la posibilidad de presentar diabetes aumenta un 17% a medida que aumenta el índice de masa corporal (IMC) (OR 1,17; IC95% 1,03-1,34; p=0,01). Finalmente, las mujeres fueron más propensas a presentar HTA (OR 3,71; IC95% 1,01-13,72; p=0,04) y CIC (OR 3,43; IC95% 1,06-11,10; p=0,03).

Conclusión: el aumento en la edad, el sexo femenino y el NI medio y bajo son factores y condiciones de vulnerabilidad que predisponen un aumento en la prevalencia de CM en adultos de San Luis, Argentina.

## Abstract (en)

The prevalence of obesity depends on biopsychosocial and environmental factors and represents a risk factor for communicable and non-communicable diseases. Objectives: To determine the association between demographic, socioeconomic and lifestyle characteristics and the presence of obesity and its metabolic comorbidities (MC) in adults in San Luis City, Argentina.

Observational population-based cross-sectional study of 306 individuals aged 18-85 years from San Luis, Argentina, selected by multistage random sampling, with an overweight prevalence of 35% and a 0.05 margin of error. Socioeconomic, demographic, and lifestyle variables were assessed, and multiple logistic regression models were fitted with the presence of obesity and MC as outcomes and sociodemographic and lifestyle characteristics as covariates.

Obesity was found in 17.3% of participants, diabetes in 3%, high blood pressure (HBP) in 11%, dyslipidemia in 3.3% and coronary ischemic complications (CIC) in 13%. The proportion of residents with at least one of these conditions was 26.8%. Low Educational level (EL) was positively associated with the presence of obesity (OR 3.58; IC95% 1.04-12.24; p=0,04), and its MC (OR 5.25; IC95% 1.05-26.23; p=0.04) with respect to high EL. Similarly, the possibility of presenting CIC was increased in people with medium EL (OR 5.8; IC95% 1.12-30.19; p=0.03). On the other hand, the possibility of presenting diabetes increased by 17% with increasing body mass index (BMI) (OR 1.17; IC95% 1.03-1.34; p=0.01). Finally, women were more likely to present HBP (OR 3.71; IC95% 1.01-13.72; p=0.04) and CIC (OR 3,43; IC95% 1,06-11,10; p=0,03).

Conclusion: the increase in age, female sex and medium and low NI are factors and conditions of vulnerability that predispose an increase in the prevalence of MC in adults from San Luis, Argentina.

## Resumo

A prevalência da obesidade depende de fatores biopsicossociais e ambientais e é um fator de risco para doenças transmissíveis e não transmissíveis. Objetivo: determinar a associação entre características demográficas, socioeconômicas e de estilo de vida e a presença da obesidade e suas comorbidades metabólicas (CM) em adultos na cidade de San Luis, Argentina.

Estudo observacional-populacional de corte transversal em 306 indivíduos entre 18 e 85 anos de idade de San Luis, Argentina, selecionados por amostragem aleatória de múltiplos estágios, considerando uma prevalência de excesso de peso de 35% e um erro de 0,05. Foram investigadas variáveis socioeconômicas, demográficas, de estilo de vida e demográficas, e foram ajustados modelos de regressão logística múltipla, incluindo a presença de obesidade e a CM como variável de resposta, e características sociodemográficas e de estilo de vida como covariáveis.

A obesidade foi encontrada em 17,3% dos participantes, o diabetes em 3%, a hipertensão arterial (HTA) em 11%, a dislipidemia em 3,3% e as complicações isquêmicas coronarianas (CIC) em 13%. A proporção de habitantes com pelo menos uma dessas patologias foi de 26,8%. O baixo NI (Nível de instrução) foi positivamente associado à presença de obesidade (OR 3,58; IC95% 1,04-12,24; p=0,04), e seu CM (IC95% 1,05-26,23; p=0,04) em relação ao alto NI. Da mesma forma, a chance de desenvolver doença cardíaca foi aumentada em pessoas com NI médio (OR 5,3; IC95% 1,12-30,19; p=0,03). Por outro lado, a chance de desenvolver diabetes aumentou em 17% com o aumento do índice de massa corporal (IMC) (OR 1,17; IC95% 1,03-1,34; p=0,01). Por fim, as mulheres tinham maior probabilidade de desenvolver hipertensão (OR 3,71; IC95% 1,01-13,72; p=0,04) e CIC (OR 3,43; IC95% 1,06-11,10; p=0,03).

Conclusão: o aumento da idade, o sexo feminino e o NI médio e baixo são fatores e condições de vulnerabilidade que predispõem ao aumento da prevalência do CM em adultos de San Luis, Argentina.

**CONCEPTOS CLAVE**

**Qué se sabe sobre el tema.**

La obesidad es una enfermedad crónica que representa un importante reto para los sistemas de salud debido a su alta prevalencia, costo de tratamiento y relación con enfermedades no transmisibles. Su aumento se debe a factores como la transición nutricional, los cambios en patrones dietéticos y el sedentarismo. Los grupos sociales de menores ingresos y con menor nivel educativo presentan mayores índices de obesidad. Los factores ambientales son clave en la definición y propuestas de intervención en las enfermedades no transmisibles.

**Qué aporta este trabajo.**

En este estudio se ha encontrado que factores demográficos y socioeconómicos están relacionados tanto con el desarrollo de obesidad como con sus complicaciones. Estos hallazgos pueden ser útiles para desarrollar políticas preventivas específicas que reduzcan las disparidades socioeconómicas en la prevención de enfermedades crónicas. Este estudio puede ser una herramienta para orientar las políticas sanitarias en términos individuales y comunitarios, y se sugiere profundizar en los factores sociales y contextuales para generar acciones claras para controlar y disminuir la prevalencia de estas patologías en el futuro.

**Divulgación**

Este estudio fue realizado para conocer la relación entre las condiciones socioeconómicas y demográficas, y la presencia de obesidad, así como sus complicaciones metabólicas en adultos. La importancia de este estudio radica en el hecho de que la obesidad es un problema de salud que aumenta el riesgo de otras enfermedades, incluyendo diabetes, enfermedades coronarias, hipertensión arterial, entre otras.

Se estudiaron 306 personas de la Ciudad de San Luis, Argentina, y se recopiló información sobre su edad, sexo, nivel de educación, socioeconómico y de actividad física, ingesta energética, índice de masa corporal (IMC), y si padecían diabetes, hipertensión arterial, dislipemia y/o complicaciones isquemico coronarias (CIC). Los resultados indicaron que a mayor edad y menor nivel de instrucción incrementa la posibilidad de presentar obesidad y comorbilidades metabólicas, y ser mujer aumenta la posibilidad de padecer hipertensión arterial y cardiopatías.

Condiciones socioeconómicas desfavorables asociadas a la presencia de obesidad y sus comorbilidades metabólicas en adultos de San Luis, Argentina

## Introducción

Las enfermedades no transmisibles (ENT), con alta incidencia e impacto en la mortalidad son un desafío para los sistemas de salud. Además, ellas generan costos elevados en términos de hospitalización, tratamiento y rehabilitación ^1^ . Particularmente, la obesidad es una enfermedad crónica multifactorial debido a la compleja asociación con aspectos biológicos, psicosociales, conductuales y ambientales ^2, 3^ ; que resulta en un balance energético positivo; es decir, cuando la ingesta energética de la dieta supera el gasto ^4^ . A nivel mundial, la prevalencia de obesidad se ha duplicado en los últimos 40 años, y actualmente, más del 60% de la población está afectada ^5^ . En estos resultados influye la transición nutricional y los cambios en los patrones dietéticos, el estilo de vida, el nivel de actividad física y el acceso económico ^6^ . En Argentina, el 67,9% de los adultos tiene exceso de peso, de ellos 33,9% presentan obesidad con una significativa diferencia según el nivel de ingresos. Los grupos sociales de menores ingresos presentan mayores índices de exceso de peso, la prevalencia de obesidad fue un 21% mayor en el quintil de ingresos más bajo respecto del más alto ^7^ . La carga de obesidad se relaciona, además, de forma inversa con el nivel educativo, siendo las poblaciones en situación de vulnerabilidad socioeconómica las que presentan mayor frecuencia de esta patología ^8^ . El contexto socioambiental en donde la población se desarrolla también influye sobre el comportamiento epidémico de esta patología, y está relacionado con los hábitos alimentarios, con las distintas culturas y estilos de vida de las diferentes regiones nacionales ^9^ .

Las sociedades son cada vez más propensas al sedentarismo y hábitos alimentarios caracterizados por un incremento de comidas fuera del hogar, de alimentos ultraprocesados, y un aumento en el tamaño de las porciones. Esto, sumado a una escasa ingesta en cantidad y variedad de frutas y hortalizas, resulta en un modelo dietético caracterizado por un mayor contenido calórico y menor densidad de nutrientes, siendo estos factores claves e influyentes en el rápido desarrollo de la pandemia de obesidad y de sus comorbilidades metabólicas ^2, 10^ . Es conocido que el nivel de inflamación de bajo grado en pacientes obesos actúa como gatillo para el desarrollo de otras patologías en pacientes genéticamente susceptibles. Dada la complejidad que presentan las ENT respecto a su tratamiento y prevención, sumado a su creciente prevalencia, los factores ambientales han adquirido un mayor poder explicativo, ocupando un lugar estratégico en su definición y en las propuestas de intervención ^11^ .

Actualmente se conoce poca evidencia sobre la influencia de las características sociales sobre el comportamiento epidemiológico de estas patologías en urbes de pequeña densidad poblacional como lo es la ciudad de San Luis. Esto, sumado a la naturaleza epidémica de la obesidad en un complejo y cambiante contexto social y ambiental, es que resulta de interés determinar la asociación entre las características sociodemográficas individuales y del estilo de vida, con la presencia de obesidad y (diabetes, hipertensión arterial (HTA), dislipidemias y CIC).

## Materiales y Métodos

### Muestreo y recolección de datos

Se realizó un estudio observacional, de corte transversal analítico poblacional y correlacional en la ciudad de San Luis en el año 2016. Esta localidad tiene un tamaño poblacional de 169.947 habitantes, según los últimos datos censales en 2010 ^12^ . En esa ciudad, se extrajo una muestra de 306 sujetos mediante un diseño de muestreo aleatorio multietápico, dividiendo estratégicamente a la ciudad de San Luis en cuatro zonas: noreste, noroeste, sureste y suroeste. Dentro de cada zona se seleccionaron aleatoriamente los barrios, según su estrato socioeconómico, considerando el criterio de homogeneidad dentro de cada estrato. Así, se identificaron un total de 126 barrios, y en cada uno de ellos, fueron seleccionadas aleatoriamente las manzanas, y dentro de ellas, entre 2 y 3 viviendas de manera sistemática, encuestando una sola persona por vivienda. Para el cálculo muestral se partió de una prevalencia de referencia de sobrepeso del 35%, según datos de prevalencia de estudios previos ^13-14^ , con un margen de error de 0,05.

La muestra estuvo constituida por adultos mayores de 18 años de ambos sexos, residentes de la ciudad de San Luis. Fueron excluidos: mujeres gestantes o en periodo de lactancia, o bien sujetos que por razones religiosas o enfermedad siguieran dietas especiales, así como individuos cuya condición mental le imposibilitaba responder.

Para la obtención de los datos se realizó una entrevista presencial a cada participante, previa firma de un consentimiento informado. En esta instancia se utilizó una encuesta que recopiló información sociodemográfica, bienes, servicios y características de la vivienda de los individuos. El nivel socioeconómico (NSE) fue construido según la Cámara Argentina de Marketing considerando el tipo de vivienda, servicios y bienes con los que contaban (computadora, conexión a internet, tarjeta de crédito, y automóviles), cobertura médico sanitaria, nivel de escolaridad alcanzado por el jefe de familia y número de integrantes asalariados. Así, el NSE quedó conformado en tres categorías: bajo, medio, alto. Posteriormente, en función de los niveles de estudios alcanzados, el NI fue categorizado en tres: bajo si incluía hasta el nivel primario completo; medio, si alcanzaba el nivel secundario; y alto si tenía estudios superiores, completos o incompletos.

Además, se indagó sobre antecedentes personales de salud, incluyendo presencia de dislipemia, diabetes, CIC e HTA. Por otra parte, se realizaron mediciones antropométricas (peso, talla, circunferencia de cintura) bajo protocolo estandarizado ^15^ . Se calculó con estos datos el Índice de Masa Corporal (IMC), para clasificar a los sujetos en las categorías de bajo peso (IMC <18,5 km/m^2^), normopeso (IMC 18,5 – 24,9 kg/m^2^), sobrepeso (IMC > 25kg/m^2^) el que incluye preobesidad (IMC 25-29.5 kg/m^2^) y obesidad (IMC >30 kg/m^2^). Seguidamente, para conocer la información sobre el consumo alimentario se usó un cuestionario de frecuencia alimentaria (FFQ) con 127 alimentos, adaptado y validado ^15^ . A cada participante se le pidió que indicara la frecuencia de consumo de cada alimento, teniendo en cuenta su consumo habitual durante el último año, y para identificar el volumen de la porción se usó un atlas fotográfico de alimentos con imágenes ilustrativas en tres tamaños (pequeño, mediano y grande) ^16^ . Por último, se calculó el consumo promedio de los alimentos (g/día) y nutrientes (g, mg o mcg/día) para cada sujeto encuestado, utilizando el software Nutrio ^15^ . Para indagar sobre el nivel de actividad física (METs/día -Unidades de Índice Metabólico por minuto y semana-) Se utilizó el International Physical Activity Questionnaire (IPAQ) en su versión corta.

El presente trabajo cumple con los principios éticos de la Declaración de Helsinki y sus modificaciones y cuenta con aprobación del Comité de Ética de la Universidad Nacional de San Luis bajo Resolución de Consejo Superior 179/14.

### Análisis estadístico

Primeramente, se observó la distribución de las variables y de acuerdo a la naturaleza de los datos se aplicaron los test estadísticos correspondientes. Se usaron pruebas de chi cuadrado o de probabilidad exacta de Fisher, considerando estadísticamente significativas las pruebas de hipótesis con valores de p<0,05.

Posteriormente, se ajustaron modelos de regresión logística múltiple para estimar la asociación entre las variables de interés, analizando como variables dependientes la presencia/ausencia de obesidad y/o CM en su conjunto, así como cada una de ellas de manera individual (obesidad, diabetes, HTA, CIC o dislipemia). Las covariables incluidas fueron edad en años, categorizado en jóvenes (18 - 26), adultos (27 - 59) y mayores (60 o más), según criterios actuales de clasificación de la Organización Mundial de la Salud.), sexo (masculino-femenino), nivel de instrucción por categoría según máximo nivel de estudios formales alcanzado (bajo–medio–alto), NSE (bajo–medio–alto), valor energético total (VET) diario aportado por la dieta (kcal/día), y el nivel de actividad física (<600, 600-1500, >1500 METs-min/semana). El análisis estadístico se realizó con el software STATA v.15.

## Resultados

La muestra estuvo compuesta por 306 adultos entre 18 y 85 años, de los cuales un 64,7% eran mujeres. Fueron excluidos 6 (1,6%) sujetos por presentar datos inconsistentes o faltantes en las principales variables de estudio. La edad en promedio fue de 36 (±17) años (18-85). La mayor proporción de personas eran jóvenes de entre 18 y 27 años (44,4%) y aquellos adultos de 60 años o más constituían el 15,7% de la muestra. Se registró un 65,4% de participantes pertenecientes al NSE medio, en tanto los de nivel bajo era el 20,3%. Sólo un 6,9% de la muestra tenía un nivel bajo de instrucción y el 50% se clasificó en el nivel más alto (Tabla 1).

**Tabla N° 1:** Distribución de características sociodemográficas y estilos de vida de los sujetos participantes según la presencia de comorbilidades metabólicas asociadas a la obesidad (n=306)

	Total n (%)	Obesidad y/o CM n(%)		Obesidad n(%)		Diabetes n(%)		Hipertensión n(%)		Dislipidemia n(%)		CIC n(%)		
		No	Si	No	Si	No	Si	No	Si	No	Si	No	Si
Sexo		p = 0,182		p = 0,926		p = 0,901		p = 0,057		p = 0,722		p = 0,030 *		
Femenino	108 (35,3)	84 (77,8)	24 (22,2)	89 (82,4)	19 (17,6)	105 (92,2)	3 (2,8)	105 (97,2)	3 (2,8)	101 (93,5)	7 (6,5)	100 (52,6)	8 (7,4)
Masculino	198 (64,7)	140 (70,7)	58 (29,3)	164 (82,8)	34 (17,2)	191 (96,5)	7 (3,5)	192 (97)	6 (3)	171 (86,4)	27 (13,6)	166 (83,8)	32 (16,2)
Grupos de edad (años)		p < 0,001 **		p < 0,001 **		p = 0,396		p < 0,001 **		p < 0,001 **		p < 0,001 **		
Jovenes (<27)	136 (44,4)	122 (89,7)	14 (10,3)	125 (91,9)	11 (8,1)	136 (100)	0 (0)	134 (98,5)	2 (1,5)	135 (99,2)	1 (0,8)	134 (98,5)	2 (1,5)
Adultos (27-59)	122 (39,9)	88 (72,1)	34 (27,9)	95 (77,9)	27 (22,1)	119 (97,5)	3 (2,5)	117 (95,9)	5 (4,1)	115 (94,2)	7 (5,7)	113 (92,6)	9 (7,4)
Mayores (>60)	48 (15,7)	14 (29,2)	34 (70,8)	33 (68,7)	15 (31,2)	41 (85,4)	7 (14,6)	46 (95,8)	2 (4,2)	22 (45,8)	26 (54,2)	19 (35,6)	29 (60,4)
Nivel Socioeconomico		p = 0,026 *		p = 0,038 *		p = 0,287		p = 0,04 *		p = 0,3670		p = 0,770		
Bajo	62 (20,3)	38 (61,3)	24 (30,7)	46 (74,2)	16 (25,8)	53 (95,2)	3 (4,8)	62 (100)	0 (0)	50 (80,6)	12 (19,4)	49 (79)	13 (21)
Medio	200 (65,4)	149 (74,5)	51 (25,5)	166 (83)	34 (17)	193 (96,5)	7 (3,5)	193 (96,5)	7 (3,5)	180 (90)	20 (10)	176 (88)	24 (12)
Alto	44 (14,4)	37 (84,1)	7 (15,9)	41 (93,1)	3 (6,8)	44 (100)	0 (0)	42 (95,4)	2 (4,6)	42 (95,5)	2 (4,5)	42 (93,2)	3 (6,8)
Nivel de instrucción		p < 0,001 **		p < 0,001 **		p = 0,127		p < 0,001 **		p = 0,02 *		p < 0,001 **		
Bajo	21 (6,9)	6 (28,6)	15 (71,4)	11 (52,4)	10 (47,6)	19 (90,5)	2 (9,5)	19 (90,5)	2 (9,5)	14 (66,7)	7 (33,3)	12 (57,1)	9 (42,9)
Medio	130 (42,5)	85 (65,4)	45 (34,6)	102 (78,5)	28 (21,5)	123 (94,6)	7 (5,4)	128 (98,4)	2 (1,5)	107 (82,3)	23 17,7)	104 (80)	26 (20)
Alto	155 (50,6)	133 (85,8)	22 (14,2)	140 (90,3)	15 (9,7)	154 (99,4)	1 (0,6)	150 (96,8)	5 (3,2)	151 (97,4)	4 (2,6)	150 (96,8)	5 (3,2)
Ingesta calórica (kcal/d)		p < 0,001 **		p < 0,001 **		p = 0,127		p < 0,001 **		p = 0,02 *		p < 0,001 **		
Tercil 1 (<2590)	105 (34,3)	73 (69,5)	32 (30,5)	87 (82,9)	18 (17,2)	100 (95,2)	5 (4,5)	103 (92,1)	2 (1,9)	92 (87,6)	13 (12,4)	87 (82,9)	18 (17,1)
Tercil 2 (<3689)	104 (34)	78 (75)	26 (25)	86 (82,7)	18 (17,3)	101 (97,1)	3 (2,9)	100 (96,1)	4 (3,9)	93 (89,4)	11 (10,6)	93 (89,4)	11 (10,6)
Tercil 3 (>3689)	97 (31,7)	73 (75,3)	24 (24,7)	80 (82,5)	17 (27,5)	95 (97,9)	2 (2,1)	94 (96,9)	3 (3,1)	87 (89,7)	10 (10,3)	86 (88,7)	11 (11,3)
Actividad Física 1		p = 0,022 *		p = 0,264		p = 0,753		p = 0,057		p = 0,169		p = 0,038 *		
< 600	102 (33,3)	65 (63,7)	37 (36,3)	80 (78,4)	22 (21,6)	96 (94,1)	6 (5,9)	98 (96,1)	4 (3,9)	85 (83,3)	17 (16,7)	82 (80,4)	20 (16,6)
600 – 1500	71 (23,2)	53 (74,6)	18 (25,4)	58 (81,7)	13 (18,3)	69 (97,2)	2 (2,8)	69 (97,2)	2 (2,8)	63 (88,7)	8 (11,3)	62 (87,3)	71 (12,7)
> 1500	133 (43,5)	106 (79,7)	27 (20,3)	115 (86,5)	18 (13,5)	131 (98,5)	2 (3,2)	130 (97,7)	3 (2,3)	124 (93,2)	9 (11,1)	122 (91,7)	133 (8,3)

1 Miliequivalentes metabólicos (METs) por minuto minuto por semana | CIC: Complicaciones isquémicas coronarias

* p <0,05 ** p <0,001

La prevalencia de sobrepeso entre los participantes del estudio fue del 48,37% y de los cuales el 17,32% presentó obesidad. Para las CM de interés la proporción fue del 3% para diabetes, 3,27% para dislipidemia, 11,11% hipertensión y 13% CIC (figura 1). El 26,8% de los participantes presentó al menos una de las patologías estudiadas, lo que afectaba al 22,2% de los varones, y al 29,3% de las mujeres.

**Figura N° 1:**
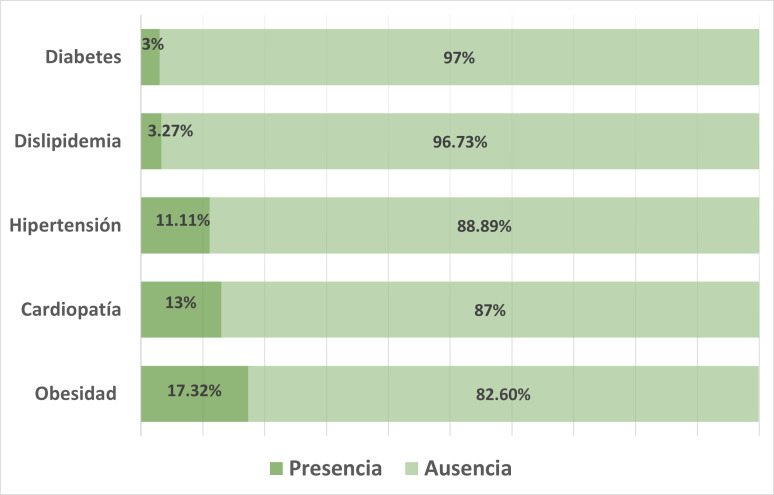
Prevalencia de obesidad y comorbilidades metabólicas asociadas en adultos de la Ciudad de San Luis, Argentina (n=306).

En relación a características del estilo de vida, un 45,3% del total de participantes tenían un nivel alto de actividad física, mientras que 23,2% realizaban niveles moderados de actividad, quedando un 33,3% de individuos sedentarios. El consumo energético medio fue de 3148 kcal/día (DE 1180) (tabla 1).

Se observó que más del 70% de los mayores de 60 años padecían al menos una de las afecciones estudiadas, mientras que, en adultos entre 27 y 59 años, el 28% presentaba alguna de estas patologías. La edad también se asoció con la presencia de obesidad, dislipemia, HTA y cardiopatía individualmente (p<0,01).

El NSE y el nivel de instrucción se asociaron con la presencia de las patologías estudiadas al analizarlas en conjunto, así como con la presencia de obesidad y HTA al evaluar estas patologías individualmente (p<0,005). Además, el nivel de instrucción se asoció con la presencia de dislipemia y de cardiopatía (p<0,001). Como se observa en la tabla I, a menor NSE y nivel de instrucción, mayor fue la prevalencia de las afecciones en conjunto e individualmente, con excepción de la HTA.

El nivel de actividad física se asoció con la presencia de cardiopatía (p=0,03) y de obesidad y con la presencia de CM en conjunto (p=0,02), siendo más frecuente la presencia de estas afecciones en personas que presentaron niveles de actividad física bajo (Tabla 1).

Los resultados del ajuste de modelos de regresión logística múltiple, se observan en la figura 2. Tener un nivel de instrucción medio aumentó la probabilidad de presentar CIC, respecto al nivel alto (OR 5,3; IC95% 1,12-30,19; p=0,03). Asimismo, un bajo nivel de instrucción se asoció positivamente con la presencia de obesidad (OR 3,58; IC95% 1,04-12,24; p=0,04), lo cual se repite con el resto de comorbilidades (p<0,05). La posibilidad de presentar diabetes aumenta un 17% a medida que aumenta el IMC (OR 1,17; IC95% 1,03-1,34; p=0,01). Por otro lado, las mujeres presentaron 3 veces más la posibilidad de tener HTA (IC95% 1,01-13,721,01p=0,04) y CIC (IC95% 1,06-11,10; p=0,03).

**Figura N° 2:**
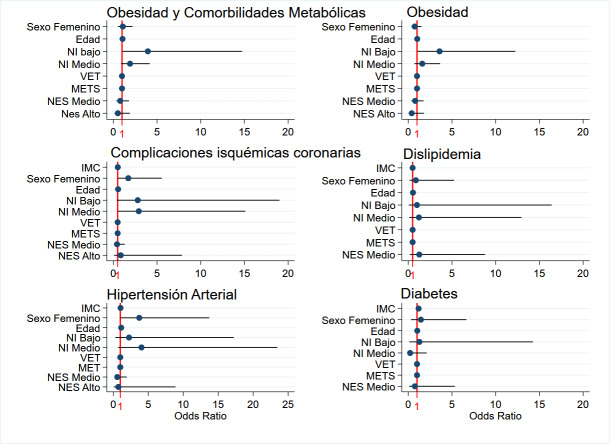
Estimación de los valores de odds ratio ajustados y sus intervalos de confianza para la presencia de obesidad y comorbilidades metabólicas en conjunto (modelo 1), obesidad (modelo 2), diabetes (modelo 3), hipertensión arterial (modelo 4), CIC (modelo 5) y dislipidemia (modelo 6), en relación a características sociodemográficas y del estilo de vida. Nota: la línea horizontal indica el odds ratio 1, es decir ausencia de asociación entre los valores estudiados.

La edad se asoció positivamente con las patologías estudiadas, excepto con diabetes (dislipemia: OR 1,08-p<0,001; cardiopatía: OR 3,4-p<0,001; hipertensión: OR 1,1-p<0,001; obesidad: OR 1,02-p=0,004). El consumo energético diario, nivel de actividad física, y NSE, no se asociaron con la presencia de estas patologías.

## Discusión

En el presente estudio la presencia de obesidad fue del 17,32% y del total de la muestra estudiada, más de un cuarto de los participantes presentó al menos una de las condiciones estudiadas. El nivel de instrucción se vio fuertemente asociado con la presencia de estas condiciones, lo que indica un aumento en la posibilidad de padecerlas a medida que se tienen NI más bajos. El IMC se asoció positivamente con la presencia de diabetes. La edad de los participantes se asoció positivamente a la ocurrencia de todas las enfermedades estudiadas y las mujeres tuvieron más posibilidades de padecer HTA y CIC.

El presente estudio muestra una alta prevalencia de sobrepeso y obesidad, así como la presencia de CM en distintas proporciones. La asociación con el sexo femenino de la presencia de cardiopatía e HTA se asemeja a lo que ocurre en países desarrollados, donde la primera causa de muerte en mujeres se atribuye a la presencia de CIC, cuyo mayor factor de riesgo concomitante es la HTA ^17-19^ . Un estudio reciente en mujeres hospitalizadas en la ciudad de Córdoba, concluye en que esta asociación puede tener relación con la escasa evidencia sobre factores de riesgo y fisiopatología de las CIC en mujeres ^17^ . Es posible, que las diferencias en el manejo terapéutico y pronóstico entre hombres y mujeres sea debido otras variables que influyen como el perfil de riesgo inicial, la percepción de dicho riesgo y las influencias hormonales propias de cada sexo ^18^ . Si bien la morbilidad por género resulta mixta, es importante considerar que, dentro de un grupo con las mismas condiciones socioeconómicas, los individuos pueden tener diversas dinámicas de comportamiento o metabolismo, lo que puede condicionar la mayor o menor vulnerabilidad de acuerdo al sexo, para desarrollar alguna patología. Un ejemplo de esto lo indica la asociación de la alteración lipídica con el sexo, donde se observó una mayor concentración de colesterol sérico en mujeres respecto de los varones ^20^ .

La edad también resultó ser un factor importante respecto a la prevalencia de dislipidemias y obesidad, en concordancia con un estudio que analizaba las CM en adultos de México, en el que encontraron que conforme aumenta la edad, más frecuente es la presencia de estas patologías, que luego de los 50 años de edad comienzan a decrecer ^20^ . Similares conclusiones sobre el efecto de la edad se hallaron en poblaciones vecinas de Argentina ^21^ .

Los resultados de este trabajo evidencian que el nivel de actividad física en más de un tercio de los participantes era insuficiente, siendo esto más frecuente en personas con al menos una enfermedad crónica y con CIC en particular. Un nivel insuficiente de actividad física tiene repercusiones negativas en el estado nutricional y de salud en general, representando el cuarto factor de riesgo en lo que respecta a la mortalidad mundial, según la OMS ^22^ . La inactividad física es reconocida como factor predictor de prácticamente todas las enfermedades que implican elevado costo sanitario y son causantes de muerte prematura ^23^ . Una exhaustiva revisión sobre la actividad física en relación a los entornos sociales y diversas patologías crónicas indica que las mujeres, adultos y adultos mayores, de bajos estratos socioeconómicos, y nivel educativo nulo y bajo, además de una mala percepción de su salud general, son quienes tienen mayor probabilidad de ser inactivos y de tener una baja autoeficacia (confianza en la capacidad de ser físicamente activo en situaciones específicas) ^24^ . Además, la evidencia científica indica que, al aumentar el nivel de actividad física, se tiende a disminuir significativamente el peso corporal, la tensión arterial, la glucemia y constituye un factor que mejora el estado general, reduciendo la morbimortalidad que implican estas patologías ^24, 25^ .

En relación a las características sociodemográficas, una amplia mayoría de los participantes de este estudio (79%) pertenecían al NSE medio y alto, y en su totalidad residían en zona urbana. Del mismo modo, más del 93% eran individuos con niveles medio y superior de instrucción. Esto se corresponde con un informe realizado por el Ministerio de Hacienda de la Provincia de San Luis, donde indicó que San Luis registró el cuarto porcentaje del país más alto de trabajadores ocupados con estudios secundarios (72%), en tanto que casi un 20% había finalizado estudios superiores ^26^ .

Resulta interesante mencionar las características de urbanización y condiciones socioeconómicas en que conviven los participantes del estudio, ya que al relacionarlo con un estudio llevado a cabo en una población similar mostró que los habitantes de las zonas urbanas tienen en promedio una mayor probabilidad de desarrollar alguna de estas patologías, que los habitantes de las zonas rurales ^27^ . El NSE en este estudio se vio asociado con la presencia de obesidad, HTA, así como con todas las comorbilidades en conjunto. En cuanto al bajo nivel de instrucción, la asociación más clara fue con la presencia de obesidad, dislipidemia, HTA, cardiopatía, y la ocurrencia de CM en conjunto. Resultados de un estudio poblacional de similares características en la ciudad de Córdoba durante 2005-2009 concuerdan con los presentados en este estudio ^21^ . Estudios similares a nivel nacional, concuerdan con dar relevancia a los factores sociodemográficos en su papel en los procesos de transición nutricional y de salud de los argentinos, conformando los actuales perfiles de riesgo, y que tal como se evidencia, la mayoría de estos se relacionan con una carga de obesidad, y por ende mayor riesgo de desarrollo de sus CM ^28^ .

Estudios epidemiológicos previos reportaron una asociación inversa entre el nivel socioeconómico y de instrucción tanto con la ocurrencia de estas afecciones crónicas, como con sus reconocidos factores de riesgo tales como un mayor IMC, deficiente calidad de la alimentación, entre otros ^27, 29^ . Las personas con bajo nivel educativo pueden tener menos acceso a información sobre hábitos de vida saludables y pueden estar en mayor riesgo de exposición a factores de riesgo de ECNT. Identificar estas relaciones es fundamental para comprender cómo abordar las ECNT de manera efectiva, particularmente en personas en situación socioeconómica más desfavorecida.

Un estudio llevado a cabo en Estados Unidos estimó un total de 145.243 muertes atribuibles a una baja educación. Las repercusiones sociales de la educación son considerables: una mejor formación está ligada a un mejor trabajo, y por consiguiente, a una mayor remuneración. El hecho de hablar de mayor ocurrencia de enfermedades o muertes atribuibles a un bajo nivel educativo subraya la relevancia del concepto "salud en todas las políticas". Esto implica el reconocimiento de que prácticamente todos los aspectos de la vida tienen el potencial de ejercer un impacto en la salud de la población humana. Los resultados significativos de nuestras estimaciones subrayan la imperiosa necesidad de considerar la educación como un componente esencial de las políticas de salud pública.

Así es como con los resultados de este estudio, queda en evidencia que la mejora en los niveles educativos de la población, además de constituir un derecho humano fundamental, puede ser una acción favorable en el intento por disminuir la tasa de prevalencia de enfermedades crónicas no transmisibles. Es posible que un mayor nivel de instrucción se vincule con una respuesta frente a la enfermedad más eficiente, el paciente tenga una mayor adherencia a los tratamientos y a las pautas terapéuticas, aunque otros aspectos socioculturales podrían complejizar más el escenario. Esto permitiría mayor efectividad en la intervención a la hora de decidir sobre sus hábitos de salud considerando, además, las consecuencias que trae consigo la presencia de estas patologías, siempre que las condiciones socioeconómicas y políticas ofrezcan un entorno favorable para ello. Similares conclusiones fueron desarrolladas a raíz de un estudio poblacional en México, que sugiere que bajo ciertas circunstancias (buena educación, un mínimo nivel de ingreso, disponibilidad de información para el cuidado de la salud) es posible transitar hacia estilos de vida más saludables que beneficien a la mayoría de la población ^30^ .

Este estudio tiene algunas limitaciones a ser consideradas en su interpretación. En primer lugar, cabe mencionar el hecho de que las afecciones estudiadas fueron autorreportadas, en lugar de ser diagnosticadas por personal médico. Considerando esto, la encuesta indagaba información adicional que permitía tener una mayor veracidad en las respuestas (por ejemplo, medicación tomada, medición de presión arterial, etc). Por otro lado un mayor volumen muestral podría dar resultados aún más confiables. Pese a las dificultades dadas por la realización de la encuesta domiciliaria, la estrategia de muestreo de base poblacional aleatorio y multietápico utilizada garantizó una alta representatividad de la población estudiada, alcanzando un número de participantes adecuado. Esto último, sumado a que este tipo de estudio es el primero en llevarse a cabo en la ciudad de San Luis, representan las fortalezas del presente artículo.

En conclusión, los resultados de este estudio en la muestra estudiada indican que los factores sociales estuvieron asociados tanto con la presencia de obesidad, como con sus CM. Estos hallazgos pueden ser útiles en la planificación de políticas preventivas específicas para reducir las disparidades socioeconómicas a fines de atender lineamientos de prevención primaria de enfermedades crónicas. Es posible que deban tenerse en cuenta otras características además de las consideradas en este trabajo, en investigaciones futuras, así como una muestra poblacional más amplia o diseños de estudios de cohorte que puedan brindar evidencias más precisas sobre estas asociaciones.

Este estudio puede aportar información valiosa con miras a orientar planificaciones de políticas sanitarias en el contexto presentado, no sólo en términos individuales sino comunitarios, donde convergen las variables sociodemográficas y socioeconómicas analizadas. Puede significar de este modo, una herramienta de acción en la vigilancia epidemiológica, siendo factible de reproducir en otros contextos similares. al de la población estudiada.

## References

[1] González Rodríguez R, Cardentey García J. Comportamiento de las enfermedades crónicas no transmisibles en adultos mayores. Revista Finlay [Internet]. 2018 ;8(2):103–110.

[2] Mohammadbeigi A, Asgarian A, Moshir E, Heidari H, Afrashteh S, Khazaei S, Ansari H. Fast food consumption and overweight/obesity prevalence in students and its association with general and abdominal obesity. J Prev Med Hyg. 2018 Sep 28;59(3):E236-E240. doi: 10.15167/2421-4248/jpmh2018.59.3.830. 10.15167/2421-4248/jpmh2018.59.3.830 PMC6196377 30397681

[3] Jehan S, Zizi F, Pandi-Perumal SR, McFarlane SI, Jean-Louis G, Myers AK. Energy imbalance: obesity, associated comorbidities, prevention, management and public health implications. Adv Obes Weight Manag Control. 2020;10(5):146-161. PMC7725222 33305001

[4] Chooi YC, Ding C, Magkos F. The epidemiology of obesity. 2019 Mar;92:6-10. doi: 10.1016/j.metabol.2018.09.005. 10.1016/j.metabol.2018.09.005 30253139

[5] Reddy KS. Global Burden of Disease Study 2015 provides GPS for global health 2030. Lancet. 2016 Oct 8;388(10053):1448-1449. doi: 10.1016/S0140-6736(16)31743-3. 10.1016/S0140-6736(16)31743-3 27733278

[6] Jiwani SS, Carrillo-Larco RM, Hernández-Vásquez A, Barrientos-Gutiérrez T, Basto-Abreu A, Gutierrez L, Irazola V, Nieto-Martínez R, Nunes BP, Parra DC, Miranda JJ. The shift of obesity burden by socioeconomic status between 1998 and 2017 in Latin America and the Caribbean: a cross-sectional series study. Lancet Glob Health. 2019 Dec;7(12):e1644-e1654. doi: 10.1016/S2214-109X(19)30421-8. Erratum in: Lancet Glob Health. 2020 Mar;8(3):e340. 10.1016/S2214-109X(19)30421-8 PMC7613084 31708145

[7] Ministerio de Salud y Desarrollo Social. Presidencia de la Nación. Encuesta Nacional de Nutrición y Salud. Banco de Recursos de Comunicación del Ministerio de Salud de la Nación. 2° Encuesta Nacional de Nutrición y Salud. Ministerio de Salud y Desarrollo Social. Presidencia de la Nación. Secretaría de Gobierno de Salud. Cobertura Universal de Salud. 2019. Disponible en: https://bancos.salud.gob.ar/sites/default/files/2020-01/encuesta-nacional-de-nutricion-y-salud-2019.pdf

[8] GBD 2016 Risk Factors Collaborators. Global, regional, and national comparative risk assessment of 84 behavioural, environmental and occupational, and metabolic risks or clusters of risks, 1990-2016: a systematic analysis for the Global Burden of Disease Study 2016. Lancet. 2017 Sep 16;390(10100):1345-1422. doi: 10.1016/S0140-6736(17)32366-8. Erratum in: Lancet. 2017 Oct 14;390(10104):1736. Erratum in: Lancet. 2017 Oct 28;390(10106):e38. 10.1016/S0140-6736(17)32366-8 PMC5614451 28919119

[9] Swinburn BA, Sacks G, Hall KD, McPherson K, Finegood DT, Moodie ML, Gortmaker SL. The global obesity pandemic: shaped by global drivers and local environments. Lancet. 2011 Aug 27;378(9793):804-14. doi: 10.1016/S0140-6736(11)60813-1. 10.1016/S0140-6736(11)60813-1 21872749

[10] Quintero Y, Bastardo G, Angarita C, Cordova JGR, Suarez CI, Uzcategui A. El estudio de la obesidad desde diversas disciplinas. Múltiples enfoques una misma visión. Venez. Endocrinol. Metab. 2020;18(3):95–106.

[11] Arnaiz MG, Demonte F, Kraemer FB. Prevenir la obesidad en contextos de precarización: respuestas locales a estrategias globales. Salud colectiva. 2020; 16:81–82. doi: 10.18294/sc.2020.2838. 10.18294/sc.2020.2838 33374091

[12] Instituto Nacional de Estadística y Censos de la República Argentina. Censo 2010. Disponible en: https://www.indec.gob.ar/indec/web/Nivel4-Tema-2-41-135

[13] Filozof C, Gonzalez C, Sereday M, Mazza C, Braguinsky J. Obesity prevalence and trends in Latin-American countries. Obes Rev. 2001 May;2(2):99-106. doi: 10.1046/j.1467-789x.2001.00029.x. 10.1046/j.1467-789x.2001.00029.x 12119667

[14] Serra Majem L, Aranceta Bartrina J. Nutrición y salud pública: métodos, bases científicas y aplicaciones. Barcelona: Mason; 2006. p. 199-210.

[15] Peyrano M, Gigena J, Muñoz SE, Lantieri M, Eynard AR, Navarro A. A computer software system for the analysis of dietary data in cancer epidemiological research. En: 17th International Cancer Congress Bologna: Monduzzi Editore. 1998. p. 381–384.

[16] Navarro A, Cristaldo P, Díaz M, Eynard A. Atlas fotográfico de de alimentos para cuantificar el consumo de alimentos y nutrientes en estudios nutricionales epidemiológicos en Córdoba, Argentina. Rev de la Fac. de Cs. Méd. de Córdoba. 2000;57(1):67–74. Disponible en: https://revistas.unc.edu.ar/index.php/med/article/view/37280 11188856

[17] García Bello L, Cáceres C, Gómez N, Paniagua M, Lovera O, Centurión OA. Factores de riesgo y cardiopatías prevalentes en mujeres internadas en la división de medicina cardiovascular del hospital de clínicas. Mem Inst Invest Cienc Salud (Impr). 2017 ;45–55. doi: 10.18004/mem.iics/1812-9528/2017.015(02)45-055.

[18] Mosca L, Benjamin EJ, Berra K, Bezanson JL, Dolor RJ, Lloyd-Jones DM, Newby LK, Piña IL, Roger VL, Shaw LJ, Zhao D, Beckie TM, Bushnell C, D'Armiento J, Kris-Etherton PM, Fang J, Ganiats TG, Gomes AS, Gracia CR, Haan CK, Jackson EA, Judelson DR, Kelepouris E, Lavie CJ, Moore A, Nussmeier NA, Ofili E, Oparil S, Ouyang P, Pinn VW, Sherif K, Smith SC Jr, Sopko G, Chandra-Strobos N, Urbina EM, Vaccarino V, Wenger NK; American Heart Association. Effectiveness-based guidelines for the prevention of cardiovascular disease in women--2011 update: a guideline from the American Heart Association. J Am Coll Cardiol. 2011 Mar 22;57(12):1404-23. doi: 10.1016/j.jacc.2011.02.005. Erratum in: J Am Coll Cardiol. 2012 May 1;59(18):1663. 10.1016/j.jacc.2011.02.005 PMC3124072 21388771

[19] Smith SC Jr, Blair SN, Bonow RO, Brass LM, Cerqueira MD, Dracup K, Fuster V, Gotto A, Grundy SM, Miller NH, Jacobs A, Jones D, Krauss RM, Mosca L, Ockene I, Pasternak RC, Pearson T, Pfeffer MA, Starke RD, Taubert KA. AHA/ACC Guidelines for Preventing Heart Attack and Death in Patients With Atherosclerotic Cardiovascular Disease: 2001 update. A statement for healthcare professionals from the American Heart Association and the American College of Cardiology. J Am Coll Cardiol. 2001 Nov 1;38(5):1581-3. doi: 10.1016/s0735-1097(01)01682-5. 10.1016/s0735-1097(01)01682-5 11691544

[20] Martínez-Hernández AF, Chávez-Aguirre R. Prevalencia y comorbilidad de dislipidemias en el primer nivel de atención. Rev Med Inst Mex Seguro Soc. 2007;45(5):469–475. 18294438

[21] Aballay LR, Osella AR, Celi A, Del Pilar Díaz M. Overweight and obesity: Prevalence and their association with some social characteristics in a random sample population-based study in Córdoba city, Argentina. Obes Res Clin Pract. 2009 May;3(2):I-II. doi: 10.1016/j.orcp.2009.01.001. 10.1016/j.orcp.2009.01.001 24345561

[22] Milton K, Gomersall SR, Schipperijn J. Let's get moving: The Global Status Report on Physical Activity 2022 calls for urgent action. J Sport Health Sci. 2023 Jan;12(1):5-6. doi: 10.1016/j.jshs.2022.12.006. 10.1016/j.jshs.2022.12.006 PMC9923423 36528290

[23] Lee IM, Shiroma EJ, Lobelo F, Puska P, Blair SN, Katzmarzyk PT; Lancet Physical Activity Series Working Group. Effect of physical inactivity on major non-communicable diseases worldwide: an analysis of burden of disease and life expectancy. Lancet. 2012 Jul 21;380(9838):219-29. doi: 10.1016/S0140-6736(12)61031-9. 10.1016/S0140-6736(12)61031-9 PMC3645500 22818936

[24] Muñoz-Rodríguez DI, Arango-Alzate CM, Segura-Cardona ÁM. Entornos y actividad física en enfermedades crónicas: Más allá de factores asociados. Univ salud. 2018;183–199. doi: 10.22267/rus.182002.122.

[25] Casco Palma J del C. Estilo de vida y su relación con el estado nutricional de los trabajadores del Instituto Politécnico de la Salud, UNAN-Managua. Torreón. 2019;7(18):49–57. doi: 10.5377/torreon.v7i18.7716.

[26] Ministerio de Hacienda Pública. Dirección Nacional de Asuntos Provinciales. Informe sintético de caracterización socio-productiva 2018. San Luis. Disponible en: https://www.economia.gob.ar/dnap/economica/14.Fichas_Provinciales/san_luis.pdf

[27] Balia S, Jones AM. Mortality, lifestyle and socio-economic status. J Health Econ. 2008 Jan;27(1):1-26. doi: 10.1016/j.jhealeco.03.001. 10.1016/j.jhealeco.2007.03.001 17448554

[28] Tumas N, Rodríguez Junyent C, Aballay LR, Scruzzi GF, Pou SA. Nutrition transition profiles and obesity burden in Argentina. Public Health Nutr. 2019 Aug;22(12):2237-2247. doi: 10.1017/S1368980019000429. 10.1017/S1368980019000429 PMC10260422 30859931

[29] Thio CHL, Vart P, Kieneker LM, Snieder H, Gansevoort RT, Bültmann U. Educational level and risk of chronic kidney disease: longitudinal data from the PREVEND study. Nephrol Dial Transplant. 2020;35(7):1211-1218. doi: 10.1093/ndt/gfy361. 10.1093/ndt/gfy361 30541108

[30] Córdova-Villalobos JA, Barriguete-Meléndez JA, Lara-Esqueda A, Barquera S, Rosas-Peralta M, Hernández-Avila M, de León-May ME, Aguilar-Salinas CA. Las enfermedades crónicas no transmisibles en México: sinopsis epidemiológica y prevención integral Salud Publica Mex. 2008 Sep-Oct;50(5):419-27. doi: 10.1590/s0036-36342008000500015. 10.1590/s0036-36342008000500015 18852939

